# The impact of follow-up care for patients presenting with non-typical chest pain at the emergency department

**DOI:** 10.1007/s12471-025-02009-3

**Published:** 2026-01-05

**Authors:** Leonard Voorhout, Mieneke Willems, Frank Willems, Sanne Heijmans, Jelle Luijten, Martin Hemels, Ron Pisters

**Affiliations:** 1 Department of Cardiology, Rijnstate Hospital, Arnhem, The Netherlands; 2 Department of Cardiology, Radboud University Medical Center, Radboud University, Nijmegen, The Netherlands

**Keywords:** Non-typical chest pain, Cardiac emergency department, Outpatient follow-up, Healthcare utilization

## Abstract

**Background:**

Failure to identify the underlying cause of chest pain in patients presenting to the cardiac emergency department (ED) poses a significant health and economic challenge. Non-typical chest pain in patients without a history of cardiovascular disease often leads to uncertainty regarding appropriate follow-up care.

**Research question:**

Does outpatient follow-up consultation with a cardiologist impact recurrent cardiac ED visits and major adverse cardiac and cerebrovascular events (MACCE) in patients with non-typical chest pain and no prior cardiovascular history?

**Study design and methods:**

This retrospective cohort study included 429 patients presenting to the cardiac ED with non-typical chest pain and no history of cardiovascular disease. Of these, 213 patients (49.7%) received follow-up consultations with a cardiologist within three months of their index ED visit. We compared rates of recurrent (cardiac) ED visits, MACCE, and healthcare resource utilization during a one-year follow-up between patients who received follow-up consultations and those who did not.

**Results:**

Patients with follow-up consultations had a significantly higher rate of revisits to the cardiac ED (13.6% vs. 5.1%) during the one-year follow-up period. There was no significant difference in MACCE between the two groups. Additionally, follow-up consultations were associated with an increase in healthcare resource utilization, including specialized cardiac tests.

**Conclusion:**

This study highlights the potential drawbacks of routine follow-up consultations in this patient population and calls for further prospective research to validate these findings.

**Supplementary Information:**

The online version of this article (10.1007/s12471-025-02009-3) contains supplementary material, which is available to authorized users.

## What is already known on this topic

Patients with non-typical chest pain often receive extensive cardiac testing despite low risk of major cardiac events. The effect of routine cardiology follow-up on outcomes and healthcare use is unclear.

## What this study adds

Routine cardiology follow-up after an ED visit for non-typical chest pain increases healthcare use—more ED (re)visits and testing—without reducing major cardiac or cerebrovascular events.

## How this study might affect research, practice, or policy

Findings support a more selective approach to follow-up in low-risk patients, aiming to reduce unnecessary resource use while maintaining patient safety.

## Introduction

Failure to identify the root cause of chest pain in patients who present to the (cardiac) emergency department (ED) poses a serious health economic issue. Chest pain is one of the most common complaints for which patients seek immediate medical attention [1]. Timely diagnosis and treatment are crucial in managing potentially life-threatening conditions and preventing adverse outcomes [2, 3]. Virtually all patients are therefore rushed to the nearest appropriately equipped EDs, which are frequently overcrowded [3, 4]. Half of all ED chest pain visits are, however, due to non-urgent and non-cardiovascular causes [1]. This has led to the emergence of accelerated diagnostic protocols utilizing highly sensitive troponin. Furthermore, the establishment of rapid chest pain clinics and cardiac emergency departments has facilitated the prompt admission and intervention for high-risk patients, while enabling the efficient and secure discharge of low-risk patients [5, 6]. A notable portion of patients assessed at the ED exhibit symptoms that are likely to be unrelated to cardiac pathology, as their chest pain is not typical (atypical angina or non-anginal chest pain) and accompanied by other risk-reducing factors [7]. Among these patients, a subset is offered a cardiology outpatient clinic visit following discharge from the ED. This consultation allows for a comprehensive and specialized evaluation, enabling further assessment of the patient’s cardiac health [5]. Furthermore, it holds the capacity to offer reassurance to patients, potentially reducing the number of ED revisits. However, the veracity of this assumption remains uncertain [8–10], and our aging population with increasing healthcare demands places a strain on outpatient clinic capacity [11]. Therefore, this study aims to investigate whether cardiology outpatient visits following ED presentation for non-typical chest pain reduce the incidence of recurrent (cardiac) ED presentations.

### Methods

As part of the local introduction of High Sensitivity Troponin T (hsTnT) measurements at the Rijnstate Hospital (Arnhem, the Netherlands), a prospective registry from January 1st, 2013, to December 31st, 2013 was approved by the local ethics committee [12]. This registry consisted of all consecutive patients presenting with chest pain to the cardiac ED in whom two consecutive hsTnT measurements took place, excluding patients with a (non) ST-elevation myocardial infarction ((N)STEMI) meeting the universal definition of myocardial infarction, an admission diagnosis of heart failure, atrial fibrillation (AF), or an alternative cardiovascular (e.g., pulmonary embolism, aortic dissection) or non-cardiovascular (e.g., gastric perforation, pancreatitis) diagnosis. Finally, patients who suffered a cardiovascular event (e.g., unstable angina pectoris, myocardial infarction, or heart failure) in the past month were also excluded [12].

Given our hypothesis, we limited the original registry cohort by excluding patients with typical chest pain. In this study, we use the terms typical angina, atypical angina, and non-anginal chest pain consistent with the classification of typical and atypical angina pectoris in the 2013 European guidelines (ESC) for stable coronary artery disease (CAD). Chest pain was categorized based on three criteria: (1) retrosternal pressing or constricting sensation, (2) provoked by exertion or emotional stress, and (3) relieved by rest or sublingual nitrates. Typical angina meets all three criteria, atypical angina meets two, and non-anginal chest pain meets one or none.

**Fig. 1:**
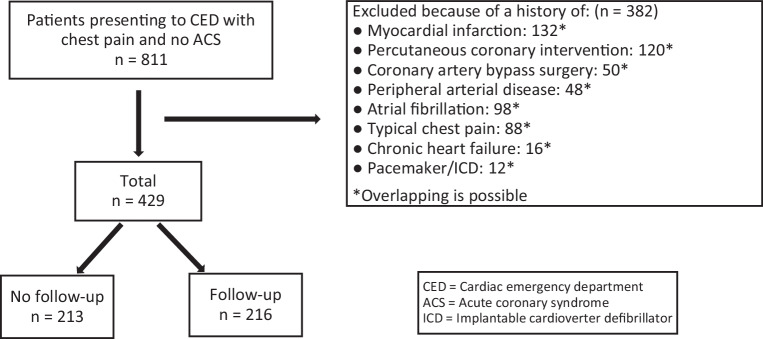
Flowchart of study cohort

Also excluded were those with a history of clinically overt atherosclerosis, i.e., a history of myocardial infarction, (percutaneous) coronary revascularization, ischemic stroke/TIA, or peripheral artery disease. Furthermore, we excluded patients with a history of AF, heart failure, presence of a pacemaker (PM) or an implantable cardioverter-defibrillator (ICD), as illustrated in Fig. 1. These additional exclusions were made as cardiology outpatient clinic follow-up visits and cardiac ED revisits could be related to these conditions, rather than their index presentation with atypical chest pain to the cardiac ED [13].

At the cardiac ED presentation, all patients underwent a clinical evaluation that included medical history, physical examination, 12-lead ECG, continuous ECG monitoring, pulse oximetry, and routine laboratory tests. HsTnT levels were measured at presentation and repeated after 3 h. The attending cardiologist had the discretion to determine if and when additional investigations and/or follow-up cardiology outpatient clinic visits should take place, which reflects real-world practice, but may also introduce selection bias.

Electronic medical records (EMR) and hospital billing data served as the primary source of data. Follow-up was conducted by performing a structured review of patient records at one year following index admission and included survival, occurrence of major adverse cardiac and cerebrovascular events (MACCE), revisits to the cardiac ED and ED, and outpatient clinic visits to the Department of Cardiology, Gastroenterology, Surgery, and/or Pulmonology. MACCE was defined as the occurrence of myocardial infarction, PCI, CABG, ischemic CVA, and/or cardiovascular or unknown death.

Hospital billing data allowed reliable retrieval of outpatient clinic consultations (following < 3 months from the index presentation) and utilization of cardiac ischemic investigations: treadmill stress testing, coronary computed tomography (CT), stress cardiac MRIs, stress echocardiography, myocardial perfusion single photon emission-computed tomography (MPS), and coronary angiograms. The outcomes of the cardiac investigations were manually retrieved by chart review and recorded as positive (e.g., significant abnormality), negative (no significant abnormality), or inconclusive (both positive and negative results or not reliable to evaluate), assessed by a cardiologist and/or radiologist, depending on the investigation. The specific criteria determining whether a result is deemed positive or negative can be found in Appendix I.

## Results

A total of 429 patients were included, with a mean age of 57 years, and 53% were female. Half of the patients received a cardiology outpatient follow-up consultation within 3 months of the index ED visit. The median time to follow-up was 47 days (IQR 38–55). The prevalence of comorbidities was notable; hypertension was present in 192 patients (45%), and hypercholesterolemia in 132 (31%). The majority of patients presented with non-anginal chest pain (62%), while 38% had atypical angina.

The HEART score was available for all patients. There was a non-significant trend toward lower HEART scores in the no-no-follow-up group compared with the follow-up group: low risk (0–3) in 74.5% vs. 66.2%, and moderate risk (4–6) in 22.5% vs. 33.8% (*p* = 0.06).

Further baseline patient characteristics, medication use at baseline, and laboratory results are described in Tab. 1. Significant differences in baseline characteristics were a higher prevalence of normal weight (BMI 18–25) (*p* = 0.010) and atypical angina (as opposed to non-anginal chest pain) (*p* = 0.007) in the group with a follow-up cardiology outpatient clinic visit. Baseline characteristics of the 382 patients excluded from the initial registry cohort can be found in Appendix II.

**Table 1:** Patient characteristics, medication and laboratory results at baseline

**Data are numbers (percentage) unless stated otherwise**	**No follow-up consultation (%) n** **=** **216**	**Follow-up consultation (%) n** **=** **213**	***P-*value**
*Female sex*	120 (56%)	106 (50%)	0.230
*Mean age, years (SD)*	56 (± 14)	58 (± 14)	0.070
*Mean BMI (SD)*	28.0 (± 5.1)	26.8 (± 4.5)	0.011
BMI 18–25	59 (27%)	83 (39%)	0.010
BMI 25–30	88 (41%)	74 (35%)	0.200
BMI > 30	62 (29%)	54 (25%)	0.435
*Comorbidities*			
Hypertension	91 (42%)	101 (47%)	0.271
Hypercholesterolemia	63 (29%)	69 (32%)	0.469
Diabetes mellitus	31 (14%)	20 (9%)	0.112
Renal insufficiency (GFR < 60 ml/min)	15 (7%)	23 (11%)	0.160
COPD	15 (7%)	11 (5%)	0.440
*Risk factors*			
Family history of cardiovasuclar disease	78 (36%)	94 (44%)	0.090
Active smoker	69 (32%)	67 (32%)	0.913
Former smoker	38/216 (18%)	49 (23%)	0.163
Non-angina chest pain	148 (69%)	119(56%)	0.007
Atypical chest pain	68 (31%)	94 (44%)	0.007
*Medication*			
Platelet aggregation inhibitors	24 (11%)	31 (15%)	0.286
Statin	39 (18%)	46 (22%)	0.358
Ace-inhibitor/ARB	43 (20%)	41 (19%)	0.864
Beta blocker	44 (20%)	48 (23%)	0.585
Calcium channel blockers	17 (8%)	18 (8%)	0.826
Nitrates	6 (3%)	8 (4%)	0.562
Diuretics	30 (14%)	32 (15%)	0.738
Proton pump inhibitors	60 (28%)	51 (24%)	0.365
*Laboratory results at baseline*	*Mean (SD)*	*Mean (SD)*	***P****-value*
GFR	79 (± 14)	77 (± 14)	0.266
Cholesterol	5.0 (± 1.1)	5.2 (± 1.1)	0.046
HDL	1.3 (± 0.41)	1.3 (± 0.42)	0.971
LDL	2.9 (± 0.88)	3.0 (± 0.93)	0.145
Triglycerides	2.0 (± 1.74)	2.0 (± 1.44)	0.867
CRP	4.6 (± 8.7)	4.6 (± 10.4)	0.973
Troponin T (1)	9.7 (± 18.1)	8.2 (± 10.6)	0.301
Troponin T (2)	10.4 (± 19.7)	8.6 (± 9.6)	0.225

### Cardiac emergency department revisits and MACCE

Cardiac ED revisits within one year occurred more frequently among patients who received a follow-up consultation (29/213, 13.6%) compared to those who did not (11/216, 5.1%) (*p* = 0.002; OR 2.94, 95% CI 1.43–6.05), as shown in Tab. 2. The majority of revisits occurred within 3 months of the initial presentation (Appendix III). Notably, 70% (28/40) of the revisits were due to recurrent, atypical angina or non-anginal chest pain. Myocardial ischemia accounted for 10% (4/40) of revisits, while 15% (6/40) were due to other cardiac causes. Two patients presented with non-cardiac or unrelated symptoms (syncope and a non-cardiac diagnosis).

**Table 2:** Cardiac ED revisits and MACCE

	No follow-up (%) n = 216	Follow-up (%) n = 213	P‑value and OR
No cardiac ED revisit(s) (1 yr)	205 (94.9%)	184 (86.4%)	
Cardiac ED revisit(s) (1 yr)	11 (5.1%)	29 (13.6%)	0.002
			OR: 2.94; 95% CI 1.43–6.05
No MACCE (1 yr)	210 (97.2%)	205 (96.2%)	
MACCE (1 yr)	6 (2.8%)	8 (3.8%)	0.569
			OR 1.37; 95% CI 0.47–4.01

MACCE occurred in 14 patients (3.3%), with no significant difference between groups: 8 events in the follow-up group (3.8%) and 6 in the group without follow-up (2.8%) (*p* = 0.569; OR 1.37, 95% CI 0.47–4.01), as detailed in Tab. 2. Eight patients underwent coronary interventions (6 PCI, 2 CABG), with 5 PCIs performed in the follow-up group. Six patients died (1.4%) during follow-up: one cardiovascular, three non-cardiovascular, and two of unknown causes. One patient experienced an ischemic stroke, which occurred in the group without follow-up. Full MACCE-related outcomes are provided in the Electronic Supplementary Material (ESM): Appendix IV.

Excluding three patients who received follow-up only after a revisit yielded similar results for ED revisits (*p* = 0.008; OR 2.63; 95% CI 1.27–5.48). (see ESM Appendix V) A broader analysis including patients with a cardiovascular history showed consistent findings (OR 2.22, *p* < 0.001) without a difference in MACCE. (see ESM Appendix VI).

In a subgroup of patients with exclusively negative ischemia tests, cardiac ED revisits still occurred more frequently in those who had a follow-up consultation (13/111, 11.7%) than in those without (11/216, 5.1%) (*p* = 0.062; OR 2.18, 95% CI 0.95–5.03) (see ESM Appendix VII). While this difference was not statistically significant, it further supports the observed trend.

Separately from cardiac ED revisits, we analyzed presentations to the general (non-cardiac) ED within 1 year after the index visit. General ED visits occurred in 13.9% (30/216) of patients without cardiology follow-up and 13.1% (28/213) of those with follow-up. Likewise, the proportion of patients with ≥ 2 general ED visits was comparable between groups (4.2%, 9/216 vs 3.8%, 8/213).

### Healthcare utilization: specialized cardiac investigations

During follow-up, 264 cardiac investigations were performed in 207 patients (48.3%). These included 171 treadmill tests (75% negative), 35 myocardial perfusion scans (80% negative), 12 cardiac stress MRIs (92% negative), 14 coronary CTs (79% negative), 10 dobutamine stress echocardiograms (90% negative), and 20 coronary angiographies (50% negative). Nearly one in four investigated patients (49/207, 23.7%) underwent multiple tests. Most investigations (86%) occurred in the follow-up group. Full distributions and results can be found in ESM Appendix VIII.

### Route to coronary angiography

Of the 20 patients who underwent coronary angiography (CAG), 10 had positive results and 10 had negative. Among those with negative CAGs, four had no prior ischemia tests; others had conflicting or inconclusive results. Similarly, diagnostic pathways before positive CAGs varied widely, including prior negative tests or combinations of inconclusive and positive findings. A visual summary of these pathways is shown in ESM Appendix IX.

## Discussion

Our findings suggest that routine cardiology outpatient follow-up after ED evaluation for atypical angina or non-anginal chest pain may be associated with increased cardiac ED revisits, without reducing MACCE. This challenges the assumption that follow-up provides reassurance or reduces healthcare utilization in this population.

Increased revisit rates were seen even among patients with exclusively negative ischemia testing, implying that consultations may not alleviate patient anxiety or persistent symptoms. This contrasts with evidence from high-risk cardiac populations, where early follow-up reduces adverse outcomes [14, 15], and highlights the need for tailored strategies in low-risk groups.

Most revisits were for recurring non-typical chest pain, suggesting unresolved concerns rather than evolving cardiac pathology [16, 17]. Previous studies have shown that patients with non-cardiac chest pain frequently return to the ED or undergo further testing, despite favorable long-term outcomes [18, 19]. Reported early revisit rates (within 7 days) range from 4–7%.

We also saw no differences in general ED visits during follow-up, suggesting that routine cardiac follow-up does not lead to more (or less) general ED visits. The significantly higher rate of additional cardiac testing, often repeated or inconclusive, raises concerns about the efficiency and necessity of follow-up in this low-risk group. This reinforces the idea that “more care” does not always equal “better outcomes.”

Alternatively, lower-intensity follow-up strategies, such as patient information materials or virtual consultations, may offer reassurance while reducing unnecessary healthcare use. These approaches deserve further exploration, particularly in the context of personalized and cost-effective care.

Beyond follow-up strategies, several recent initiatives aim to reduce the burden on cardiac emergency care earlier in the patient pathway. For instance, Dutch studies have shown that structured pre-hospital triage and single pre-hospital troponin testing can safely lower unnecessary cardiac ED presentations [20].

### Limitations

Follow-up consultations were determined by physician’s discretion, introducing potential selection bias. Although both groups were similar in size and most measured baseline characteristics were balanced, unmeasured factors, such as physician risk perception or patient anxiety, may have influenced referral decisions. This is supported by the observed trend toward slightly higher HEART scores in the follow-up group, suggesting that clinicians may have been more inclined to refer patients with a perceived higher baseline risk.

Hospital billing data were used retrospectively, but these are generally reliable, as they are routinely used for insurance claims and hospital administration. However, we were unable to capture healthcare utilization outside of our hospital, such as visits to general practitioners (GPs) or other hospitals. It is possible that patients who did not receive follow-up at our cardiology department sought care elsewhere or were managed more frequently in primary care, which may have shifted rather than reduced overall healthcare utilization. Some outpatient consultations may have been unrelated to the index ED visit (e.g., new arrhythmias). To minimize this bias, we excluded patients with known cardiovascular disease and those with revisits before their scheduled follow-up.

Importantly, this study reflects care patterns from 2013. At that time, treadmill stress testing was more commonly used than it is today. Current guidelines now discourage routine use of treadmill ECGs for evaluating chest pain. Since then, practice has shifted towards coronary CT angiography and functional imaging. Therefore, the diagnostic approaches we observed, particularly the high number of exercise tests, may not reflect current practice, which limits generalizability.

Lastly, the retrospective nature of this study precludes causal conclusions. Prospective, randomized studies comparing protocol-directed follow-up to standard care are needed to validate these findings and explore alternative strategies for follow-up in this population.

## Conclusion

Our study suggests that routine cardiology outpatient follow-up after ruling out ACS in patients with atypical angina or non-anginal chest pain is associated with significantly more cardiac ED revisits, without a reduction in MACCE. Given the aging population and increasing demand on both emergency departments and cardiology outpatient clinics, it is essential to critically evaluate the value of routine follow-up in this group. Further prospective research is guaranteed to develop more effective, individualized strategies for managing these patients and to optimize resource use in an increasingly strained healthcare system.

## Supplementary Information

The Supplementary Information contains additional analyses and supporting material, including detailed definitions of diagnostic test outcomes, baseline characteristics of excluded patients, timing of ED revisits, full MACCE outcomes, and results of sensitivity analyses (Appendices I–IX)

## Data Availability

The data supporting this study are available from the corresponding author upon reasonable request.
