# Supplementary material for: The impact of follow-up care for patients presenting with non-typical chest pain at the emergency department
Source: Neth Heart J. 2026 Jan 5;34(2):54–9. doi: 10.1007/s12471-025-02009-3 (PMC12852522; doi:10.1007/s12471-025-02009-3)
Supplement: Supplementary file 1 — The Supplementary Information contains additional analyses and supporting material, including detailed definitions of diagnostic test outcomes, baseline characteristics of excluded patients, timing of ED revisits, full MACCE outcomes, and results of sensitivity analyses (Appendices I–IX) [file 12471_2025_2009_MOESM1_ESM.docx]

Appendix I

**Criteria for positive result**

Treadmill stress tests:

1. target heart rate at least 85% of predicted max heart rate

2a. ST depression of 1mm or more; horizontally or downsloping

or

2b. ST elevation of 1mm or more in non-Q lead

Dobutamine Stress echocardiography:

1. Development or worsening of regional wall motion abnormalities during dobutamine infusion
or
2. Significant decrease in LVEF from rest to peak stress.
or
3. Decreased myocardial thickening in response to dobutamine in one or more segments.
or
4. Reduction in endocardial excursion (motion towards the center of the left ventricle) during stress.
or
5. Onset of angina, shortness of breath, or other ischemic symptoms during the test.
or
6. Development of ischemic ECG changes, such as ST-segment depression or elevation, during dobutamine infusion.

Coronary computed tomography (CT)

1. Calcium Score >75^th^ percentile
 or
 2. Agatson Score >300

Stress cardiac MRI

1. Subendocardial or Transmural Perfusion Defect: A persistent perfusion defect during stress that is not present at rest. This defect must be subendocardial or transmural, indicating impaired blood flow to the heart muscle under stress conditions.
or
2. Transient Perfusion Defect: A perfusion defect observed only during stress and not at rest, suggesting reversible ischemia.
or
3. Development of New Regional Wall Motion Abnormalities (RWMA): Any new or worsening wall motion abnormalities observed during stress but not present at rest.
or
4. Increased End-Systolic Volume (ESV): A significant increase in the left ventricular end-systolic volume under stress conditions compared to rest, indicating impaired contractile function.
or
5. Reduced Segmental Wall Thickening: A lack of appropriate thickening of the myocardial wall during stress in one or more segments of the left ventricle, indicating ischemic segments.

Myocardial single photon emission-computed tomography (MPS)

1. Perfusion Defect: A perfusion defect that is present during stress but resolves at rest, indicating reversible ischemia. This suggests that the myocardium is receiving insufficient blood supply during stress.
or
2. Fixed Perfusion Defect: A perfusion defect present both during stress and at rest, suggesting scar tissue or previous myocardial infarction. While not indicative of active ischemia, this can correlate with non-viable myocardium
or
3. Stress-Induced Wall Motion Abnormalities: Detection of wall motion abnormalities during stress, such as hypokinesia, akinesia, or dyskinesia, not present at rest. This is less commonly assessed with MPS but can provide additional information.
or
4. Transient Ischemic Dilation (TID): TID of the Left Ventricle: An increase in the apparent size of the left ventricular cavity during stress compared to rest. This can indicate severe and extensive coronary artery disease.

Coronary angiogram

1. Coronary Artery Stenosis: Diameter reduction of 50% or greater
 or
 2. Fractional Flow Reserve (FFR): FFR ≤ 0.80
or
3. Instantaneous Wave-Free Ratio (iFR): iFR ≤ 0.89Appendix II


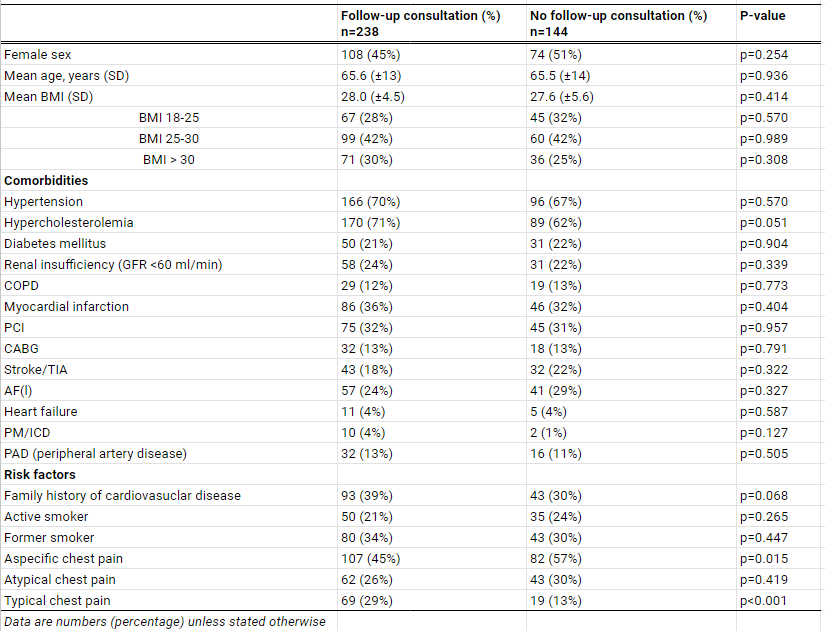


Patient characteristics at baseline of excluded patients

Appendix III

|  | **No follow-up (%)  n=216** | **Follow-up (%)  n=213** |
| --- | --- | --- |
| (first) Cardiac ED revisit (<3m after index visit) | 6 (2.8%) | 19 (8.9%) |
| (first) Cardiac ED revisit (3m-6m after index visit) | 1 (0.5%) | 4 (1.9%) |
| (first) Cardiac ED revisit (6m-12m after index visit) | 4 (1.9%) | 6 (2.8%) |
| Total (first) Cardiac ED revisit (0-1yr) | 11 (5.1%) | 29 (13.6%) |

Timing of cardiac ED revisits

Appendix IV

|  | **No follow-up consultation (%) n=216** | **Follow-up consultation (%) n=213** |
| --- | --- | --- |
| Myocardial infarction | 0 (0%) | 4 (1.9%) |
| PCI | 1 (0.5%) | 5 (2.3%) |
| CABG | 1 (0.5%) | 1 (0.5%) |
| Death | 5 (2.3%) | 1 (0.5%) |
| Cardiovascular death | 1 (0.5%) | 0 (0%) |
| *Non-cardiovascular death** | *2 (0.9%)* | *1 (0.5%)* |
| Unknown cause of death | 2 (0.9%) | 0 (0%) |
| CVA | 1 (0.5%) | 0 (0%) |
| Total unique individuals MACCE | 6 (2.8%) | 8 (3.8%) |
| * Non-cardiovascular death is not included in the MACCE calculation |  |  |

MACCE specific

Appendix V


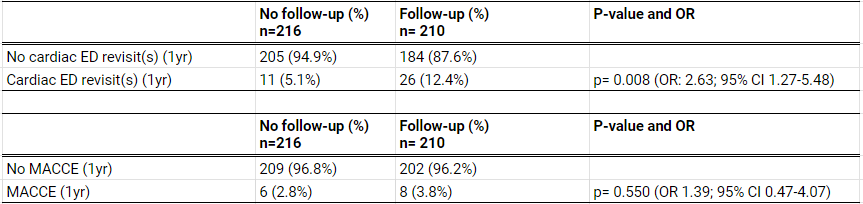


In this analysis three patients were excluded who had received an offer for follow-up consultation with a Cardiologist after a revisit to the Cardiac ED

Appendix VI


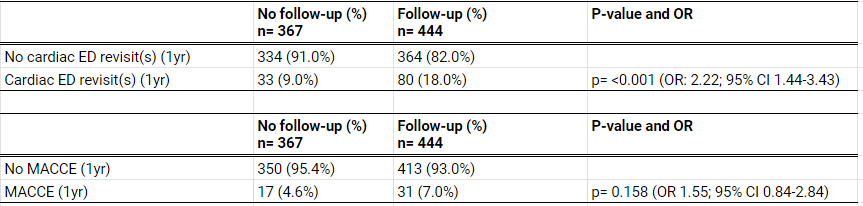


In this analysis, all (n=811) patients incorporated in the original study design are included

Appendix VII

|  | **No follow-up (%)  n= 216** | **Follow-up and exclusively negative test(s) (%)  n= 124** | **P-value and OR** |
| --- | --- | --- | --- |
| no cardiac ED revisit(s) (1yr) | 205 (94.9%) | 111 (89.5%) |  |
| cardiac ED revisit(s) (1yr) | 11 (5.1%) | 13 (10.5%) | p= 0.062 (OR: 2.18; 95% CI 0.95-5.03) |

Cardiac ED revisits in patients with exclusively negative test(s)

Appendix VIII


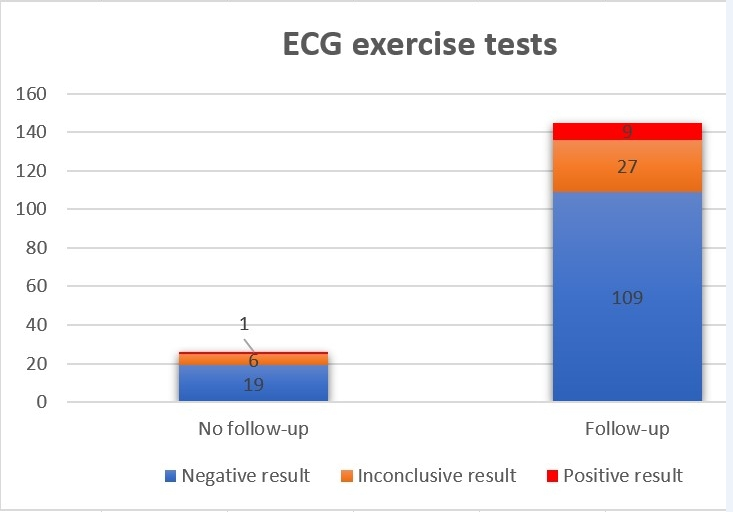


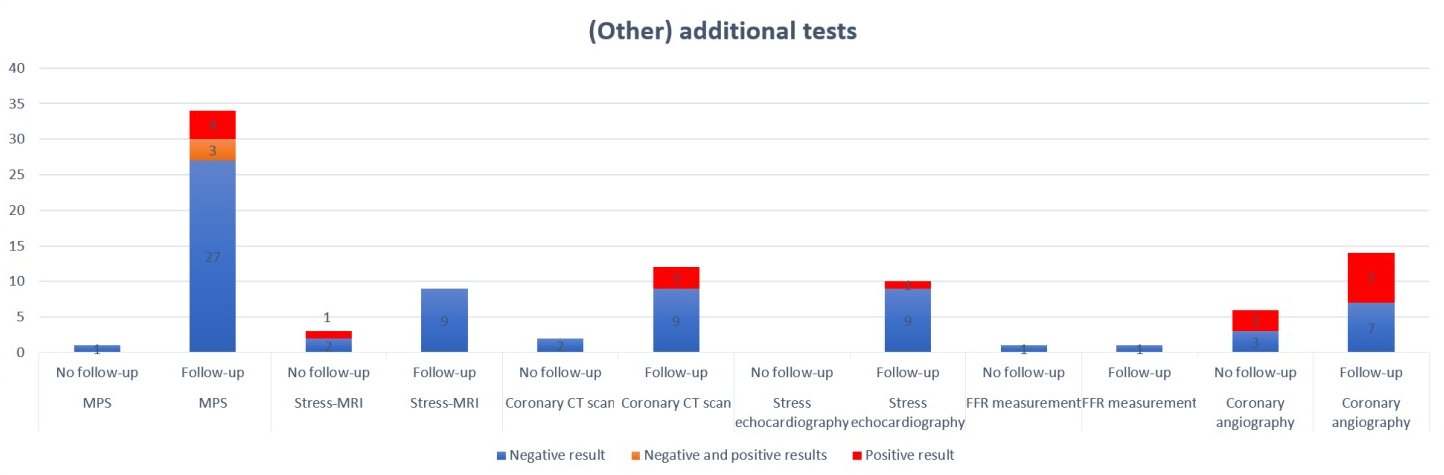


Appendix IX


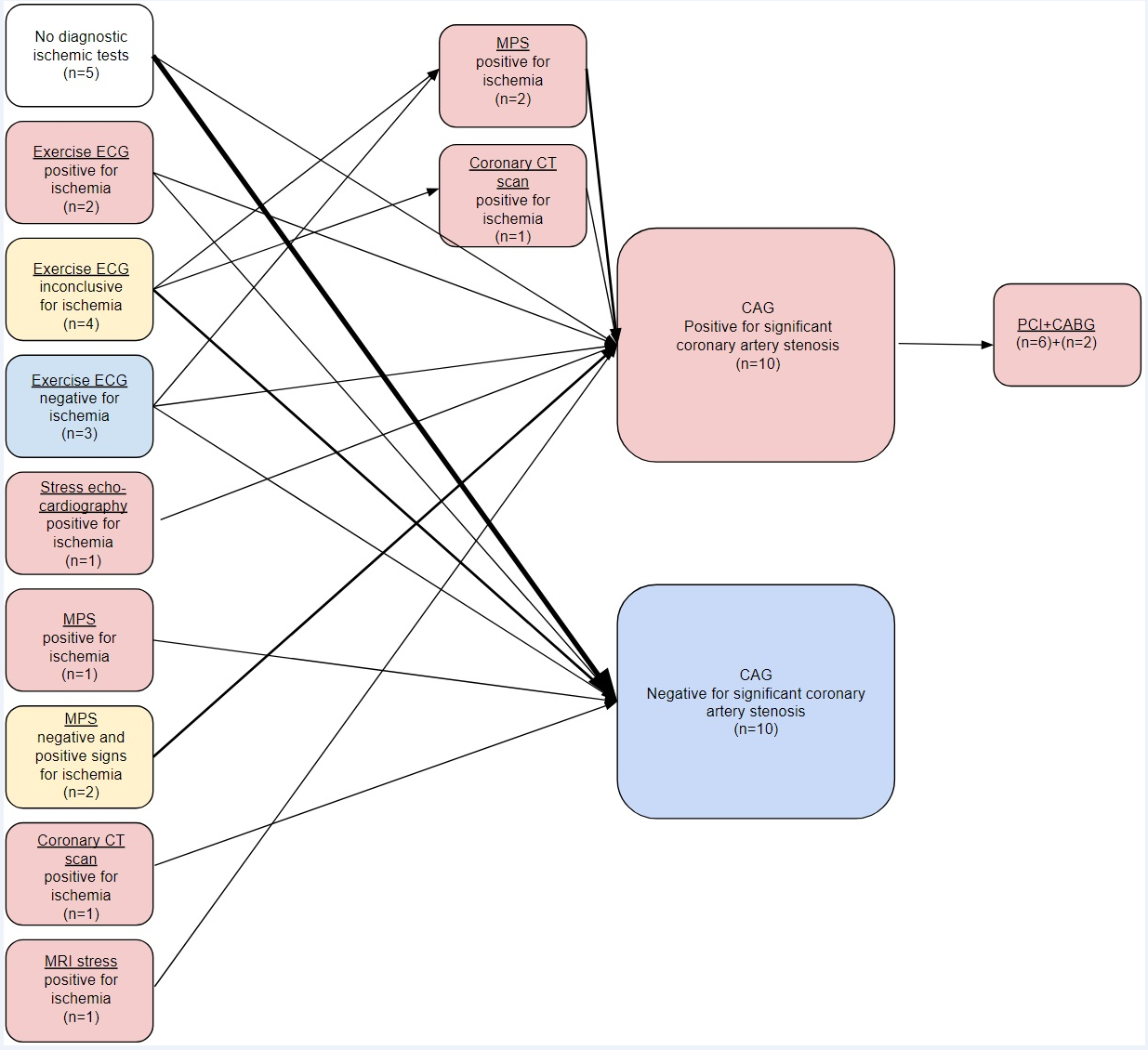


*Route to coronary angiogram (CAG)*
